# Research progress on the kidney-gut-brain axis in brain dysfunction in maintenance hemodialysis patients

**DOI:** 10.3389/fmed.2025.1538048

**Published:** 2025-03-06

**Authors:** Jie Yu, Yulu Li, Bin Zhu, Jianqin Shen, Liying Miao

**Affiliations:** ^1^Department of Nephrology, The Third Affiliated Hospital of Soochow University, Changzhou, China; ^2^Department of Nephrology, Taicang Loujiang New City Hospital, Suzhou, China; ^3^Department of Critical Care Medicine, The Third Affiliated Hospital of Soochow University, Changzhou, China; ^4^Department of Blood Purification Center, The Third Affiliated Hospital of Soochow University, Changzhou, China

**Keywords:** end-stage renal disease, maintenance hemodialysis, gut microbiota, brain dysfunction, intestinal mucosal barrier

## Abstract

Maintenance hemodialysis (MHD) has become the primary renal replacement therapy for patients with end-stage renal disease. The kidney-gut-brain axis represents a communication network connecting the kidney, intestine and brain. In MHD patients, factors such as uremic toxins, hemodynamic changes, vascular damage, inflammation, oxidative stress, and intestinal dysbiosis in MHD patients refers to a range of clinical syndromes, including brain injury, and is manifested by conditions such as white matter disease, brain atrophy, cerebrovascular disease, cognitive impairment, depression, anxiety, and other behavioral or consciousness abnormalities. Numerous studies have demonstrated the prevalence of these brain disorders in MHD patients. Understanding the mechanisms of brain disorders in MHD patients, particularly through the lens of kidney-gut-brain axis dysfunction, offers valuable insights for future research and the development of targeted therapies. This article reviews the brain dysfunction associated with MHD, the impact of the kidney-brain axis, intestinal barrier damage, gut microbiota dysbiosis caused by MHD, and the role of the gut-brain axis in brain dysfunction.

## 1 Introduction

End-stage renal disease (ESRD) is the final phase of various chronic kidney conditions and has become a significant global health and healthcare burden. In 2010, approximately 2.62 million people worldwide received renal replacement therapy (RRT), and by 2030, this number is projected to more than double, reaching 5.44 million, with Asia experiencing the fastest growth (1). Currently, the primary forms of RRT for ESRD include hemodialysis (HD), peritoneal dialysis, and kidney transplantation. In most countries, HD has become the most common treatment for ESRD patients due to its high safety and efficiency (2). However, a considerable number of ESRD patients experience brain dysfunction after receiving long-term maintenance hemodialysis (MHD). Brain dysfunction refers to clinical syndromes associated with brain injury, including consciousness disorders, somatic motor dysfunction, cognitive impairment, and mental and behavioral abnormalities, all of which seriously impact patients’ quality of life (3).

**Figure 1:**
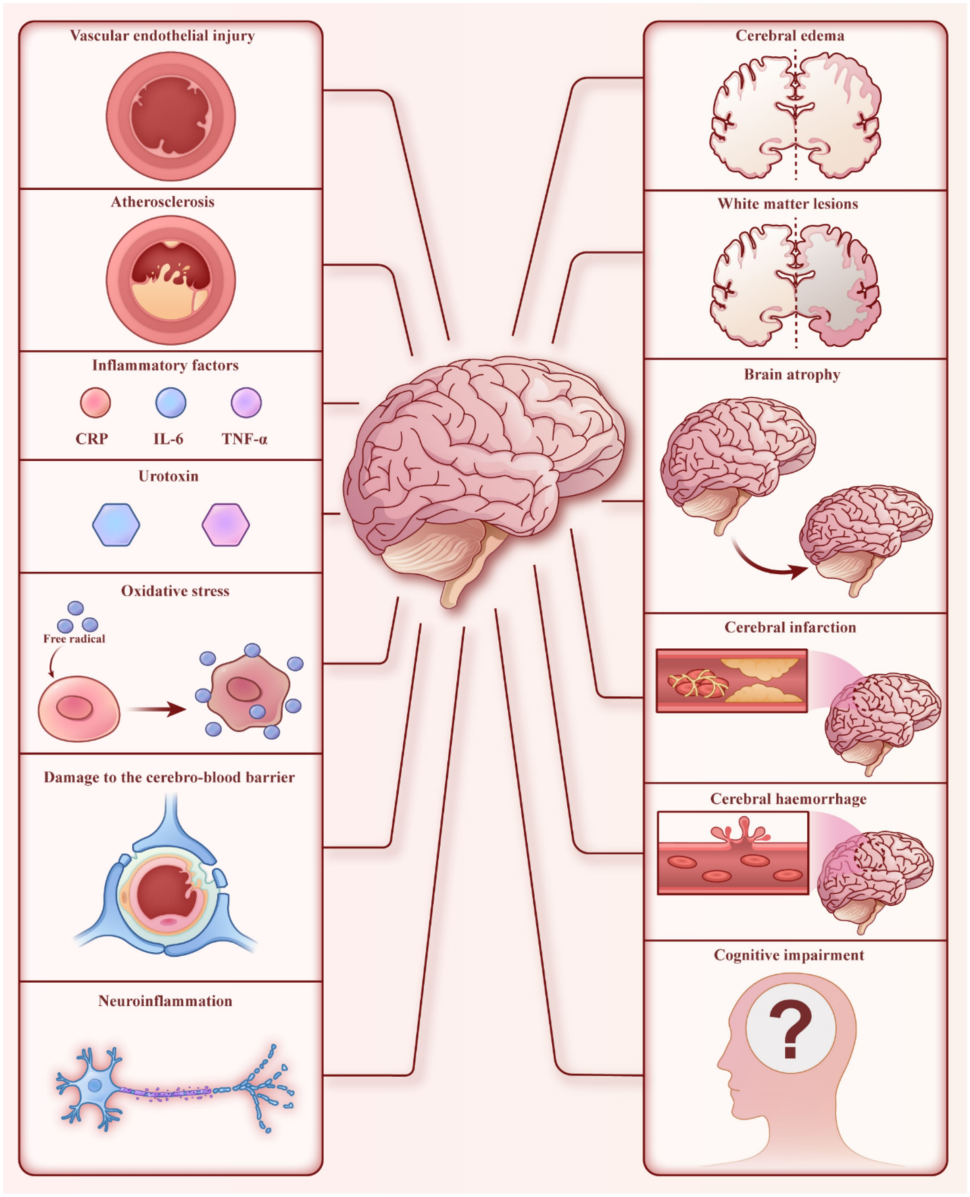
Brain dysfunction in MHD patients. The left side represents factors that may cause brain injury, while the right side shows some manifestations of brain dysfunction.

An increasing number of studies have shown that brain damage, including white matter lesions, cerebral atrophy, cerebral infarction, cerebral hemorrhage, and subclinical cerebrovascular accidents, is common in MHD patients (Figure 1) (4–10). These patients also frequently exhibit disturbances in brain functional networks (11, 12). In addition, autonomic neuropathy is often observed in MHD patients (13), contributing to significant mortality in this population. Recent studies have reported that the incidence of cognitive impairment in MHD patients ranges from 30 to 80% (14–19). Cognitive impairments in these patients affect executive function, information processing speed, language fluency, and short-term memory (14, 15, 20). Cognitive impairment is also an independent predictor of all-cause mortality in MHD patients, further increasing their mortality rate (21). In addition, epidemiological studies indicate that the prevalence of depression in MHD patients ranges from 23.7 to 52%, significantly higher than in the general population (22–24). Depression can negatively affect the quality of life in MHD patients by impacting their diet, sleep, treatment compliance, and mental state (25–28). It’s also associated with increased rates of hospitalization, cardiovascular events, and mortality risk (29, 30).

**Figure 2:**
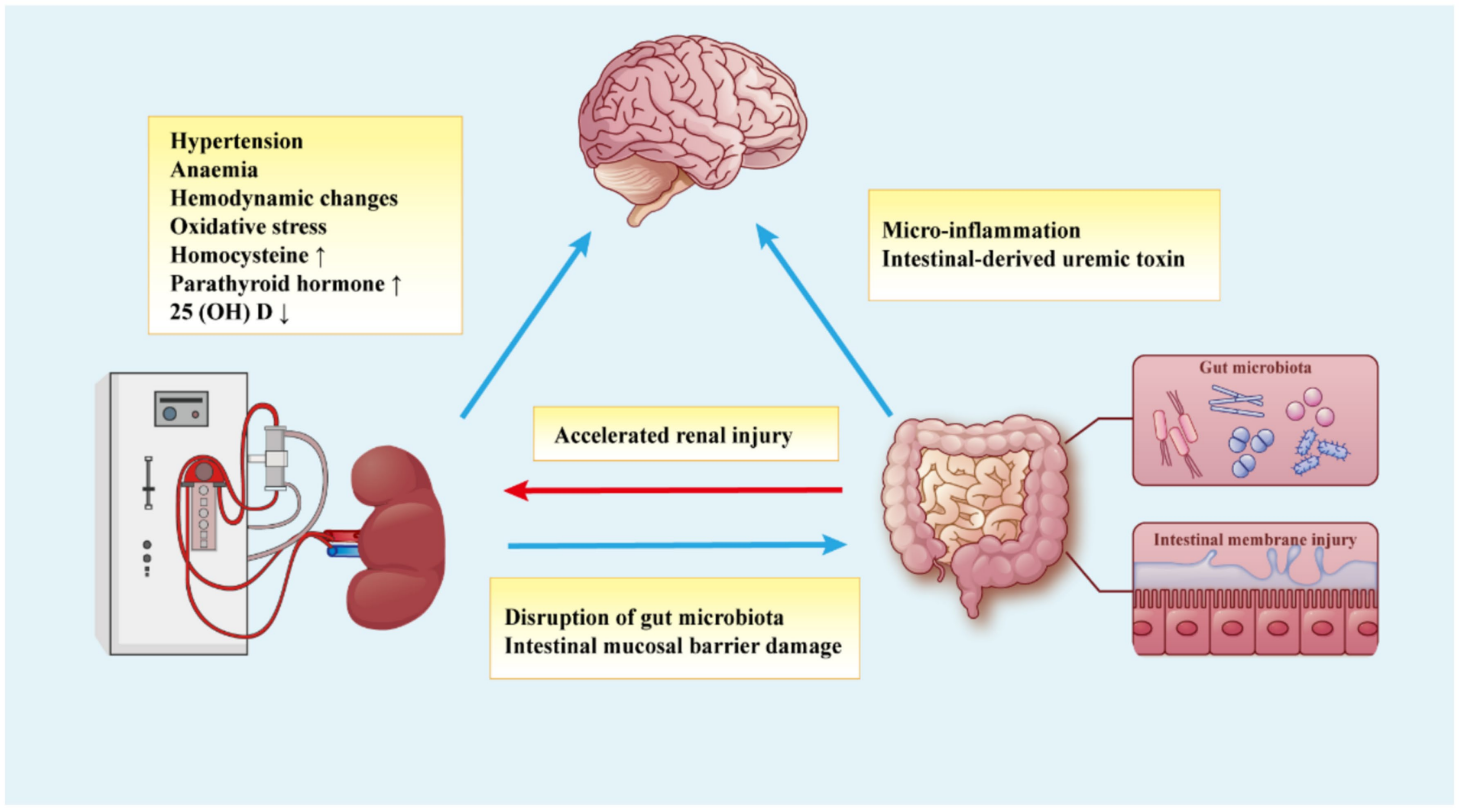
The kidney-gut-brain axis hypothesis for the mechanism of brain dysfunction in MHD patients. The brain dysfunction of MHD patients is caused by various factors, including metabolic disorders caused by renal dysfunction, dialysis-related factors, gut microbiota disorders, and intestinal mucosal damage, which constitute the communication network of the kidney-gut-brain axis.

Therefore, investigating the pathogenesis of brain dysfunction in MHD patients is crucial for improving disease treatment. In recent years, the interaction between the renal-gut-brain axis has become a key area of research. This interaction is mediated by autonomic regulation from the brain and signals from the gut and kidneys, ultimately forming the complex network of the renal-gut-brain axis (Figure 2) (31). In this review, we will explore the mechanisms underlying renal-gut-brain axis dysfunction and its role in secondary brain dysfunction in MHD patients.

## 2 The effect of the kidney-brain axis

### 2.1 Renal hypertension

Hypertension affects over 80% of patients undergoing MHD, with volume overload being a primary cause. Other contributing factors include increased activity of the renin-angiotensin-aldosterone system, sympathetic nervous system activation, endothelial dysfunction, and the use of recombinant human erythropoietin (rHuEPO) (32, 33). Accelerated arteriosclerosis in MHD patients can lead to microvascular damage, impairing the automatic regulation of cerebral blood flow (CBF) (34). The brain, due to its low vascular resistance and sustained high blood flow, is particularly vulnerable to microvascular damage caused by systemic hypertension. Elevated intravascular pressure can lead to vascular wall rupture or ischemic damage, resulting in stroke (34, 35). The risk of stroke in MHD patients is correlated with the rise in blood pressure levels (36). Yu-Huan et al. (32) found that the risk of cerebral hemorrhage increased proportionally with higher pre-dialysis systolic blood pressure in 25 MHD patients. Nearly all studies investigating the association between hypertension and cognitive decline have identified a positive correlation, likely linked to hypertension-induced white matter lesions (36). Savazzi et al. (37) performed diagnostic brain computed tomography (CT) on 25 MHD patients, all of whom were found to have cerebral atrophy. The study revealed a strong positive correlation between the degree of cerebral atrophy, pre-dialysis blood pressure values, and the duration of hypertension. The study revealed a strong positive correlation between the degree of cerebral atrophy, pre-dialysis blood pressure values, and the duration of hypertension. In a study by Qian et al. (38), brain magnetic resonance imaging (MRI) of 180 MHD patients showed that 36.1% of the HD subgroup had cerebral microbleeds (CMBs). Hypertension, duration of dialysis, and mean arterial pressure (MAP) were significantly associated with deep CMBs, all of which would impact patients’ cognitive function.

### 2.2 Renal anemia

Renal anemia is a common complication in MHD patients, with an estimated 95% requiring anemia correction therapy (39, 40). Studies have shown a significant positive linear correlation between hemoglobin (Hb) levels and local cerebral oxygen saturation (RSO_2_). Under anemic conditions, RSO_2_ decreases in MHD patients, and the subsequent reduction in oxygen delivery can impair various brain metabolic functions (41, 42). Additionally, a decrease in hematocrit (Hct) may result in increased CBF, potentially leading to the increased delivery of uremic toxins to the brain, which can cause cerebral edema (27). In a study of 56 MHD patients, Lee et al. found that patients with higher Hct levels scored better on neurocognitive function tests and demonstrated superior working memory and attention compared to those with lower Hct levels (43). Pickett et al. (44) conducted brain electrophysiological measurements and electroencephalogram spectrum analysis in MHD patients and found that brain function was significantly impaired when mean Hct was 23.7%. However, after anemia correction and an increase in mean Hct as low as 31.6%, patients showed improved attention and cognitive function. RHuEPO is commonly used to correct anemia in clinical practice. Brain event-related potentials (ERPs), which are sensitive, non-invasive, and relatively objective measures of cognitive function, have shown improvement after rHuEPO treatment. In a study evaluating ERPs in 15 MHD patients before and after rHuEPO therapy, an increase in Hct from 22.7 to 30.6% led to significant improvements in the latency and amplitude of the auditory event-related P300 peaks (45). Correcting anemia with rHuEPO treatment can improve the speed and efficiency of information processing in MHD patients, enhance attention and memory, improve cognitive function, and alleviate anxiety (45–47).

However, the use of rHuEPO is not entirely beneficial and may heighten the risk of thrombosis in MHD patients, particularly in those with diffuse atherosclerosis, thereby adversely affecting brain function (48). This may be related to increase in blood pressure, blood viscosity, and peripheral resistance. Some studies have also found that when rHuEPO is used to treat renal anemia in MHD patients, increasing Hct from 28 ± 8% to 37 ± 5% can reduce the blood flow velocity in the middle cerebral artery by 11% (48).

### 2.3 Dialysis-related factors

#### 2.3.1 Cerebral blood flow and perfusion volume

The removal of large volumes of fluid and changes in circulating blood flow during HD can reduce cerebral perfusion and CBF, often accompanied by hypoxemia. This may be one of the mechanisms by which HD causes recurrent ischemic brain injury, leading to secondary psychiatric symptoms (49, 50). The brain receives 15–20% of cardiac output due to high CBF, making it susceptible to ischemic damage, which can result in local lactic acidosis and direct neuronal toxicity (51). HD can increase Hct levels (average Hct before HD is mostly below 30%, and significantly increases after HD), which increases relative oxygen delivery capacity. As a result, less CBF is required to transport oxygen, potentially contributing to the observed decrease in mean blood flow velocity (MFV) (52). Polinder-Bos et al. (7) monitored CBF in 12 elderly patients with MHD from the beginning to the end of HD and observed that global CBF decreased by 10% ± 15%, from an average of 34.5 ml/100 g per minute to 30.5 ml/100 g. The decrease in CBF was consistent across different brain regions. In one study in 28 MHD patients, overall cognitive function in MHD patients fluctuated significantly during the dialysis cycle, with sharp cognitive declines occurring during dialysis. Cerebral blood perfusion and velocity were significantly lower after dialysis compared to pre-treatment levels, suggesting that hemodynamic changes may affect cognitive function and recovery to highest level of cognitive function 24 h after the dialysis session (49, 53).These low blood flow levels may increase the risk of transient ischemic attacks.

However, some transcranial Doppler ultrasound (TCD) measurement studies have shown that in patients not receiving rHuEPO during dialysis, cerebral perfusion significantly increased, indicating a strong negative correlation between hemoglobin levels and cerebral perfusion (54). Anemia can affect CBF velocity and volume by altering oxygen metabolism, and it can increase the cerebral oxygen extraction fraction in HD patients, potentially impairing cerebral vasodilation ability (54). In addition, the factors that cause the oscillations in cerebral perfusion in MHD patients are diverse, such as blood pH, arterial carbon dioxide, cardiac output and other factors may be important factors (50, 54). The brain, as a key perfusion organ, is particularly sensitive to circulatory changes ebb and flow of circulation (55).

Eldehni et al. reported that using dialysate at 0.5°C below core body temperature, compared to 37.0°C, improved vascular resistance, increased hemodynamic stability, and reduced the progression of white matter lesions (56). Therefore, severe brain damage caused by HD may be effectively reduced by enhancing hemodynamic tolerance through the use of cooling dialysis agents (56, 57).

#### 2.3.2 Oxidative stress

Oxidative stress (OS) is defined as an imbalance between the production of free radicals (FRs) or reactive oxygen species (ROS) and the body’s antioxidant defense system, leading to oxidative damage to cellular components and, in severe cases, cell death (58). Studies have confirmed that OS is common in MHD patients (59–61). The reasons for increased OS in MHD patients include (a) Abnormal production of oxidants, such as increased ROS production due to limited biocompatibility of HD membranes, and an increase in uremic toxins with pro-oxidative functions (58, 62); (b) Reduced absorption of food antioxidants due to malnutrition and the non-selective removal of antioxidants during dialysis, leading to a significant reduction in antioxidant level (58, 63, 64); (c) Deficiencies in antioxidant enzyme levels (61).

OS plays an important role in neurodegenerative damage and cognitive decline. Increased OS can directly induce neuronal death and intermittently reduce neuronal activation potential, thereby lowering local oxygen demand and reducing perfusion and blood supply (59). OS can impair synaptic plasticity and memory function, manifesting as cognitive decline (65). Furthermore, OS is considered a contributing factor to atherosclerotic cardiovascular disease in ESRD patients (66), although it is not related to the presence or severity of white matter lesions in the brain (60). In a study by Belaïch et al. (59), blood oxygen level-dependent brain magnetic resonance imaging (BOLD-fMRI) was performed on 86 MHD patients. The results showed that after dialysis, OS levels significantly increased systemically, compared to before dialysis. This was accompanied by a significant decrease in brain activation intensity in the motor areas and a notable increase in the volume of brain activation. These changes suggest brain plasticity induced by elevated OS levels.

#### 2.3.3 Cerebral oxygenation

Valerianova et al. (67) found that during HD, RSO_2_ in the patients’ brain decreased shortly after the start of dialysis, reaching its lowest value within 15 min. Patients with higher red blood cell distribution width (RDW) exhibited lower RSO_2_ values and greater fluctuations during HD (RDW has recently been extensively studied, and RDW elevation is associated with malnutrition, inflammation, and OS in MHD patients). Research indicates that RSO_2_ in the brain can decrease by more than 20% during HD (68), and this reduction is correlated with cognitive decline in MHD patients (69). In the study by Ookawara et al. examining the relationship between cognitive function score on mini-mental state examination (MMSE) and clinical factors in 193 MHD patients, those with MMSE scores ≥24 had significantly higher brain RSO_2_ levels (53.8% ± 8.3%) compared to patients with MMSE scores ≤23 (49.5% ± 9.8%), indicating that MMSE score is independently correlated with brain RSO_2_ (41).

#### 2.3.4 Cerebral edema

Acute loss of intravascular volume and fluid displacement during HD lead to diffuse interstitial brain edema and damage to cellular integrity, which are also associated with cognitive dysfunction (70). Walters et al. (71) conducted brain MRI scans on five patients with MHD and a normal control group, performing imaging examinations before and after HD. Brain volume changes were measured using magnetic resonance registration technology. In MHD patients, the average brain volume increased by 32.8 ml (SE 7.4 ml, accounting for 3% of brain volume) after HD, whereas the control group showed only a 1.4 ml (SE 0.6 ml) change (71). The results indicated that asymptomatic brain edema occurred in MHD patients after HD, with those experiencing the largest absolute decrease in pre-HD urea concentration showing the most brain edema. This study did not test the cognitive function of the patients. Although no patients showed significant neurological symptoms, the possibility of brain function damage in patients cannot be ruled out, and we speculate that brain volume changes will be greater in patients with neurological symptoms. Kong et al. (70) performed diffusion tensor imaging (DTI), neuropsychological (NP) tests, and laboratory tests on 80 MHD patients and a healthy control group. DTI data suggested that MHD patients had diffuse brain edema and moderate white matter integrity damage, both of which were associated with cognitive impairment. In experiments conducted on uremic dogs, Arief et al. (72) found that rapid HD was related to a decrease in cerebrospinal fluid pH and the spontaneous accumulation of osmotic pressure in the brain. This created an osmotic gradient between the brain and plasma, resulting in brain edema, increased intracranial pressure, and epileptic seizures.

There are two central hypotheses regarding the mechanism of cerebral edema, both supported by rapid HD experiments in uremic animals (73). The first hypothesis is the “reverse osmosis gradient” mechanism, in which urea is cleared from the blood faster than from the brain, creating a brain plasma osmotic gradient. The brain reacts to the higher osmotic pressure in the blood by generating its own osmotic pressure, driving water into the brain (74–76). Therefore, it is essential to adjust dialysis prescriptions carefully to minimize changes in plasma osmolality during HD treatment and reduce the risk of cerebral edema.

### 2.4 Hyperhomocysteinemia

Elevated plasma total homocysteine (THcy) levels, defined as fasting THcy levels above 1.87 mg/L (13.9 mol/L), are present in approximately 85% of HD patients, compared to about 10% in the general population (27). Elevated THcy levels can damage small arteries and are an independent risk factor for cardiovascular and cerebrovascular diseases, making THcy a novel predictor of cerebrovascular events (77, 78). Homocysteine may contribute to cognitive impairment and cerebrovascular disease through multiple mechanisms. Firstly, homocysteine directly causes neurotoxicity by inhibiting methylation reactions, increasing sensitivity to extracellular toxins, and activating the N-methyl-D-aspartate (NMDA), subtype of glutamate receptors (79–81). Secondly, homocysteine has a direct pre-thrombotic effect on the vascular system by activating platelets and reducing thrombomodulin/protein C activation, promoting blood clots formation (27, 78, 79). Thirdly, elevated homocysteine can induce oxidative stress and DNA damage in vascular endothelial cells while also increasing platelet-endothelial adhesion and promoting vascular smooth muscle cell proliferation (78, 79). Fourthly, elevated homocysteine is a reliable indicator of vitamin B12 deficiency, which can lead to neurological disorders including cognitive impairment (81). Finally, high THcy levels are strongly associated with atherosclerotic diseases, as homocysteine enhances the autoxidation of low-density lipoprotein cholesterol and increases the binding of lipoprotein A and fibrin, promoting atherosclerosis (79, 81–83). Cerebral ischemia caused by atherosclerosis may accelerate the progress of cognitive dysfunction and brain atrophy at the same time (82). Anan et al.’s study showed that THcy levels were higher in MHD patients with spinal cord injury (the most common form of subcortical cerebral infarction) compared to MHD patients without spinal cord injury (78). Additionally, Maesato et al. (79) conducted brain MRI examinations on 34 MHD patients and found that the average hippocampal atrophy rate was 27.3% and the average total brain atrophy rate was 11.2%. The study also showed a significant correlation between hippocampal atrophy and hyperhomocysteinemia in HD patients. Since the hippocampus is supplied by the anterior choroidal artery, a vessel prone to thrombotic cerebral infarction, hyperhomocysteinemia may contribute to hippocampal damage and related cognitive impairment (79).

### 2.5 Secondary hyperparathyroidism and 25 (OH) D deficiency

Secondary hyperparathyroidism (SHPT) is characterized by elevated levels of parathyroid hormone (PTH), parathyroid hyperplasia, calcium and phosphorus metabolism disorders, as well as clinical conditions such as renal bone disease and vascular calcification. On the one hand, elevated PTH can directly induce neurotoxic effects, leading to cognitive impairment and mental disorders; on the other hand, high PTH can activate parathyroid hormone 2 receptors (PTH2R), which are widely distributed in the central nervous system, affecting various neuroendocrine functions (84, 85). Diskin et al. (85) evaluated the psychological health scores using the Kidney Disease Quality of Life (KDQOL-36) and Patient Health Questionnaire (PHQ-2) on 10 patients with intact parathyroid hormone (iPTH) levels above 1,000 pg.ml and 10 MHD patients with iPTH levels below 400 pg/ml. They found that patients in the high PTH group had higher levels of depression. Other studies have shown that after parathyroidectomy in MHD patients with SHPT, brain electrical abnormalities and patients’ mental states, as well as mild central nervous system dysfunction, are improved (86). A large-scale study involving 65,849 MHD patients demonstrated that high levels of iPTH were significantly associated with an increased risk of hemorrhagic stroke and identified iPTH as a risk factor for stroke in MHD patients (87, 88). This may be due to high serum PTH levels causing changes in blood pressure, increased vasoconstriction ability, vascular smooth muscle hypertrophy, vascular calcification, fibrosis, and affecting the cerebrovascular system by promoting inflammation (32). PTH also influences the nervous system through calcium regulation mechanisms. In a study by Tanaka et al. on the relationship between mineral metabolism abnormalities and mental health in MHD patients, it was found that patients with a corrected calcium level of 11 mg/dl had significantly lower psychological health scores compared to those with corrected calcium level of <8.4 mg/dl (89). In clinical practice, MHD patients often take calcium-containing phosphate binders and active vitamin D preparations to treat SHPT (90, 91). However, during the treatment, MHD patients are prone to developing hypercalcemia, which can cause psychoneurotic symptoms (84). Elevated PTH levels also promote calcium accumulation in brain tissue, significantly increasing brain Ca^2+^ content, inhibiting mitochondrial oxidation, and reducing ATP production (86, 89, 92).

25-hydroxyvitamin D [25(OH)D] is the main circulating form of vitamin D, and deficiency of 25(OH)D due to kidney injury is common in MHD patients. Vitamin D nuclear receptors are widely expressed in the spinal cord and brain, and vitamin D plays a role in regulating the synthesis of neurotransmitters such as acetylcholine and catecholamines (84, 93). In addition, 25 (OH) D exhibits vascular protection, neuroprotection, inhibition of pro-inflammatory factor inhibition, and antioxidant and immune regulatory properties. Low levels of 25 (OH) D are associated with vascular disease risk factors, including vascular calcification, endothelial dysfunction and inflammation (93–95). Therefore, 25(OH)D deficiency is considered an important risk factor for cognitive impairment and certain neurodegenerative diseases. Shaffi et al. (95) analyzed 255 MHD patients and found that lower levels of 25(OH)D were associated with an increased likelihood of impaired executive function in the cognitive domain. Demet In another study, Yavuz et al. (94) conducted questionnaire assessments on 121 MHD patients using the Pittsburgh Sleep Quality Index (PSQI) and Beck Depression Inventory (BDI), and found that low 25(OH)D levels and high BDI values are independent risk factors impairing sleep quality. Other studies have found a significant correlation between low serum 25(OH)D levels and higher depression scores (84).

## 3 The kidney-gut axis

Since Ritz introduced the concept of “gut kidney syndrome” at the International Dialysis Conference in 2011 (96), the connection between the intestine and kidneys has been extensively studied, leading to the development of the “gut-kidney axis” theory (Figure 2). In this interaction, uremia affects the composition and metabolism of the gut microbiota, while dysbiosis of the gut microbiota leads to increased uremic toxins and microinflammation, further promoting the progression of kidney disease (97).

### 3.1 MHD causes disruption of gut microbiota in patients

The gut microbiota plays several critical roles, including energy generation, nutrient metabolism, immune regulation, and maintaining the structural integrity of the intestinal mucosal barrier (98–100). In patients with reduced renal filtration capacity, the accumulation of urea in the blood leads to its diffusion into the intestines, where it is metabolized by bacteria to produce ammonia. This process raises the pH of the intestinal lumen, disrupting intestinal homeostasis (101). The Human Microbiome Project has provided comprehensive data on 2,172 microbial species isolated from humans, divided into 12 different bacterial phyla. Of these, 93.5% belong to *Proteobacteria*, *Firmicutes*, *Actinobacteria*, and *Bacteroidetes* (102). Over 90% of healthy individuals’ gut microbiota is dominated by two main bacterial phyla, Bacteroidetes and Firmicutes, followed by *Actinobacteria* and *Verrucomicria* (98, 103). Although the human colon also contains major pathogens such as *Campylobacter jejuni*, *Salmonella enterica*, *Vibrio cholerae*, *Escherichia coli*, and *Bacteroides fragilis*, the is relatively low (typically accounting for 0.1% or less of the entire gut microbiota) (98).

In MHD patients, the bacteria in the blood do not primarily originate from dialysate (104, 105). The main bacteria detected in the blood are also present in the intestines, and the changes in bacterial colonies in the blood closely resemble those in the gut. This suggests that the bacteria in the bloodstream may come from the gut microbiota (104). HD can alleviate intestinal toxins and improve the disruption of intestinal microbiota (106). However, MHD patients still exhibit significant changes in gut microbiota abundance (104, 106–109). Studies comparing MHD patients with healthy individuals or ESRD patients who are not on dialysis have shown that the diversity of gut microbiota species in MHD patients is significantly higher than in control groups (104, 106, 109).

Bacteroidetes, which play key roles in nutrient absorption and the maturation and maintenance of epithelial cells (110), and their diversity decreases in MHD patients (104, 107, 109). A notable feature of the gut microbiota in MHD patients is the reduction of beneficial bacteria, such as *Lactobacillus* (106–108). *Lactobacillus* and *Bifidobacterium* are mainly involved in maintaining the integrity of the intestinal mucosal barrier (108). Conversely, there is an increase in pathogenic bacteria such as *Enterobacteriaceae* (106, 108). In healthy individuals, *Proteobacteria* account for less than 1% of the gut microbiota, and a low abundance of *Proteobacteria* alongside high levels of *Bacteroides, Prevotella*, and *Ruminococcus,* indicates a healthy gut microbiota (98, 103). However, studies show that in MHD patients, the levels of *Proteobacteria* increase significantly (104, 107, 109), while *Prevotella* decreases (107) and *Ruminococcus* increases (109). *Escherichia coli* and *Enterococcus faecalis* are opportunistic pathogens that can overgrow when gut microbiota balance is disrupted, leading to intestinal dysbiosis and infections (108). In MHD patients, the levels of these two bacteria are elevated (106–108). In addition, the duodenum and jejunum of uremia patients (usually not colonized by bacteria) are extensively colonized by aerobic and anaerobic bacteria, further complicating the pathogenic effects of uremia on intestinal function (96).

### 3.2 Intestinal mucosal barrier damage and bacterial translocation in MHD patients

Under normal conditions, tight junction proteins in the intestinal epithelium form an effective barrier, preventing the penetration of harmful substances such as bacteria, bacterial toxins, digestive enzymes, and partially degraded food products (111). Uremic toxins can damage the structure and function of the intestinal barrier, particularly by depleting key protein components in the tight junctions of the colon (111). Disruption of the intestinal epithelial barrier allows endotoxins and other harmful luminal contents to enter the systemic circulation, contributing to systemic inflammation (104, 112).

The decrease in effective circulating blood volume during HD can lead to temporary insufficiency of intestinal blood supply and ischemic damage to the intestinal mucosal barrier (96). Studies have shown that one in six patients undergoing MHD for 1–2 years developed gastrointestinal bleeding, and 82% of MHD patients experienced ischemic colitis (113). These conditions increase intestinal permeability and mucosal damage, facilitating bacterial translocation and the entry of bacterial endotoxins.

Malnutrition is very common among MHD patients. Long-term dietary restrictions, combined with the loss of albumin and amino acids during dialysis, may contribute to gut microbiota imbalances, particularly in patients undergoing high-throughput dialysis (114, 115). This disrupts the stable state of the gut microbiota, which relies on food for energy metabolism, increasing the risk of bacterial translocation.

### 3.3 Intestinal environment disorder aggravates renal damage progression

Ischemia–reperfusion injury of the intestinal mucosal barrier, microinflammation caused by intestinal leakage, activation of the immune response due to dysregulation of the intestinal flora and mitochondrial dysfunction, these changes in turn accelerate kidney injury (31, 96, 116). Indoxyl sulfate (IS) is a uremic toxin produced from the breakdown of tryptophan by intestinal bacteria and is normally excreted by the kidneys. In CKD, IS accumulates, exerting pro-inflammatory effects, disrupting endothelial cell function, damaging renal tubular epithelial cells and podocytes, and leading to renal interstitial fibrosis (96, 97, 101).

## 4 The effect of gut-brain axis

The gut microbiota plays a crucial role in the normal development of brain function. A growing body of animal and clinical studies has shown that gut microbiota disorders are associated with neurological conditions such as Alzheimer’s disease, autism spectrum disorder, Parkinson’s disease, depression, and stroke (117, 118). The gut-brain axis, a bidirectional communication pathway between gut bacteria and the central nervous system, involves the central nervous system, endocrine system, and immune system, forming a network that connects the gut and brain (Figure 2) (117). In our clinical research, 30 healthy individuals and 77 MHD patients were enrolled and classified into healthy control (HC), normal cognitive function (NCF), and mild cognitive decline (MCD) groups based on evaluations using the Montreal Cognitive Assessment. Compared to the HC or NCF groups, the MCD group exhibited significant changes in gut microbiota characteristics, including α- and β-diversity, and alterations in 16 specific gut bacteria. Additionally, certain blood metabolites were altered, suggesting that MHD-related MCD may be linked to abnormal gut microbiota composition and imbalances in serum metabolites. The *Bilophila* genus, in particular, may serve as a sensitive biomarker for MCD in MHD patients (119).

### 4.1 Microinflammation caused by intestinal bacteria and brain dysfunction

Microinflammation refers to a chronic low-grade inflammatory state characterized by elevated levels of circulating pro-inflammatory cytokines, such as C-reactive protein (CRP), interleukin-6 (IL-6), and tumor necrosis factor-α (TNF-α), with CRP being a reliable objective indicator of inflammatory activity (104, 120). Most ESRD patients have bacterial ectopia and inflammation, and HD can exacerbate the micro inflammatory state to a certain extent (104, 120). Approximately 30–50% of MHD patients exhibit inflammatory reactions (105).

Intestinal bacteria and their components are involved in the micro inflammatory state observed in MHD patients (105, 121). In a study of 52 ERSD patients by Shi et al., compared with non -dialysis patients, MHD patients showed slightly higher levels of hypersensitive C-reactive protein (hs-CRP), IL-6, and plasma endotoxin in their blood. The study found that the more complex the bacterial species, the higher the levels of CRP and endotoxin in their blood, indicating a positive correlation between the bacterial load in the blood and the levels of CRP and IL-6 (104). Zhang et al. (108) compared 39 MHD patients with healthy individuals and found that MHD patients had lower levels of *Bifidobacterium* and *Lactobacillus* in the intestines but higher levels of *Escherichia coli* and *Enterococcus*, all of which contributed to the low-grade inflammatory state.

Intestinal-derived bacteria increase the micro inflammatory state of MHD patients through various mechanisms, including the accumulation of circulating endotoxins and uremic toxins produced by bacteria, a reduction of anti-inflammatory bacteria, bacterial translocation caused by intestinal mucosal barrier damage, immune dysfunction caused by CKD, and long-term high-dose antibiotic use, which further increases the resistance of *Enterococci* in the intestine (104, 108, 122).

A prospective study showed that high levels of inflammatory factors in MHD patients are closely related to vascular risk factors of atherosclerosis and cardiovascular death (123). In ESRD patients and HD patients, microinflammation increases the incidence of atherosclerosis and the risk of brain disorders (120, 124, 125). In a study by Fanadka et al., over 90% of MHD patients had some degree of intracranial arterial calcification, which was correlated with serum CRP levels, indicating that micro-inflammation contributes to the changes in vascular structure (126). Another study found that hs-CRP levels are an important risk factor for asymptomatic cerebral infarction in MHD patients (127).

Inflammation also plays a crucial role in the mechanisms underlying mental disorders. The inflammatory response system (IRS), driven by pro-inflammatory cytokines, can contribute to depression by activating the hypothalamic–pituitary–adrenal (HPA) axis and increasing serotonin and catecholamines (128). Peripheral inflammation caused by gut microbiota can lead to cerebral amyloidosis and hippocampal volume reduction and may cause neurodegeneration and cognitive impairment, playing an important role in the initiation and progression of dementia (122, 129, 130). In MHD patients, the level of the inflammatory mediator prostaglandin D2 synthase (PGD_2_S) is elevated, which may induce neuronal apoptosis and, theoretically, be involved in the progression of dialysis-induced brain disorders (131). Bossola et al. used the Beck Depression Inventory (BDI) and Hamilton Anxiety Scale (HAMA) to assess depression and anxiety in 80 patients with MHD, showing IL-6 levels were positively correlated with depression scores in multivariate analysis (132). A meta-analysis found that higher levels of IL-6 and CRP are associated with an increased risk of psychological and immunological changes, including all-cause dementia and depression; IL-1 β has been identified as an important risk factor for depressive symptoms and plays a key role in neurodegenerative changes by involving in the pathogenesis of the production and deposition of amyloid β-protein in the brain of Alzheimer’s disease (128). In a study of patients with cognitive impairment, the abundance of *Odoribacter, Butyricimonas*, and *Bacteroides* in their intestines was significantly reduced. These bacteria have strong resistance to nerve inflammation and immune regulation, revealing that chronic inflammation may link the gut microbiota characteristics to cognitive impairment (130).

### 4.2 Intestinal-derived uremic toxin and brain damage

Uremic toxins are classified based on their physical and chemical characteristics during HD clearance: (I) Small, water-soluble molecules (such as urea and uric acid), which are easily removed by HD and may not necessarily be functionally toxic; (ii) Medium molecular weight compounds such as β 2-microglobulin and leptin, which affect multiple organ systems; (iii) Protein-bound compounds, typically low molecular weight compounds such as phenols and indoles, which are difficult to remove by dialysis and can significantly enhance toxic effects in the body (133–135). Protein-bound uremic toxins produced by the gut microbiota, such as IS, p-cresol sulfate (PCS), and indole-3-acetic acid (IAA), along with guanidine compounds (GCs) derived from arginine (guanidinosuccinic acid, guanidine, and methylguanidine), are considered the main uremic toxins responsible for brain damage in uremia patients (136–142).

These uremic toxins not only directly cause neurotoxicity, impair brain synaptic function, and induce astrocyte apoptosis, but they may also have harmful indirect effects on the brain through mechanisms such as blood–brain barrier disruption, microvascular changes, endothelial dysfunction, neuroinflammation, oxidative stress, and imbalances in neurotransmitter amino acids (133–135, 137–145).

The manifestations of brain damage caused by neurotoxins include cognitive impairment, drowsiness, convulsions, coma, cerebrovascular disease, and neuropathy, which can be partially improved through HD (144, 146). Intestinal-derived uremic toxins in CKD patients can induce endothelial barrier dysfunction of cerebral vessels, leading to cerebral microbleeds (147). Secondary restless leg syndrome (RLS) is also frequently associated with various neurological disorders caused by uremic toxins (148). Among these toxins, IS has been extensively studied for its neurotoxic effects. Several animal experiments have shown that IS can increase oxidative stress and neuroinflammation, disrupt the blood–brain barrier, impair glial cell function, and induce neuronal cell death in a dose-dependent manner (143, 149). High serum IS concentrations are associated with more severe cognitive impairment (143). Lin et al. (134) evaluated 260 MHD patients based on the MMSE and the Cognitive Ability Screening Inventory (CASI), and found that after adjusting for confounding factors, circulating free IS levels were negatively correlated with MMSE and CASI scores (134).

IAA, another uremic solute from the indole family, is metabolized in tissues through serotonin and other tryptophan derivatives and is toxic to astrocytes and microglia (135). Higher plasma IAA levels in MHD patients are linked to reduced microbial diversity (150). IAA can induce endothelial dysfunction, inflammation, and oxidative stress, increasing the risk of cardiovascular damage and cognitive impairment (150). A study involving 230 MHD patients showed a significant correlation between IAA and cognitive impairment (135). IAA is also associated with anxiety and depression symptoms in CKD patients (151).

In addition, a systematic study on endotoxemia in CKD revealed that serum endotoxin levels in MHD patients were nearly six times higher than those in non-dialysis patients (152). HD-induced systemic circulation stress and repeated ischemia may increase the translocation of intestinal endotoxin and induce endotoxemia, which can lead to a wide range of adverse effects on vascular structure and function (133, 152).

### 4.3 Neurotransmitters and neural transmission pathways produced by intestinal bacteria

Recent studies have shown that gut microbiota regulates the synthesis and function of neurotransmitters such as glutamate, γ-aminobutyric acid (GABA), acetylcholine, norepinephrine, serotonin, and dopamine (153–155). Since some neurotransmitters cannot penetrate the blood–brain barrier, they must be synthesized in the brain by local neurotransmitter precursors. Therefore, changes in the abundance of gut microbes can alter the expression of neurotransmitter receptors in the brain (117, 153). The gut microbiota can also transmit sensory signals to the brain through the vagus nerve, participating in signal transmission in neural pathways. This interaction influences brain function and cognitive behavior, suggesting the role of gut microbiota in the pathogenesis of various neuropsychiatric diseases (156–158).

In early studies, two abnormalities were observed during autopsies of patients who had suffered from dialysis encephalopathy: a significant decrease in GABA levels in several brain regions (frontal, occipital, and cerebellar cortex, caudate nucleus, and medial dorsal thalamus) and a reduction in cholinergic acetyltransferase activity in the cerebral cortex (159, 160).

### 4.4 Intestinal microbiological intervention therapy

After understanding the physiological functions and pathological mechanisms of intestinal flora, many researchers have explored various methods to reconstruct healthy intestinal flora. In recent years, three dietary supplements, probiotics, prebiotics, and synbiotics, have been considered to have a significant impact on the balance of the gut microbiota (161, 162).The comprehensive analysis of several clinical trials in HD patients showed that probiotics, prebiotics and synbiotics could significantly reduce the levels of circulating toxins (PCs, endotoxin) and inflammatory biomarkers (CRP, IL-6), and improve the balance between antioxidants and pro-oxidant markers to reduce oxidative stress (163, 164). However, there is still a knowledge gap as to whether there are specific gut microbial species and their metabolites that can be used as potential therapies for treatment.

## 5 Summary and perspective

In summary, this review explores the imbalance of the kidney-gut-brain axis and elucidates the mechanisms of interaction between the three organs in secondary brain dysfunction in MHD patients. Firstly, factors such as hypertension, anemia, and metabolic abnormalities resulting from renal dysfunction in MHD patients contribute to brain damage. We discussed how HD treatment affects brain structure and function by causing decreased brain perfusion, OS, and osmotic pressure differences. Secondly, we summarized the dysbiosis of the gut microbiota and the damage to the intestinal mucosal barrier observed in MHD patients. Finally, we explained how disturbances in gut microbiota, through the gut-brain axis, lead to neuronal damage and mental disorders by inducing internal microinflammation, the accumulation of uremic toxins, and disruptions in signal transmission pathways. Although increasing studies are uncovering the complex communication between the gut, brain, and kidneys, many specific mechanisms of action remain unclear. Future research could focus on identifying potential biomarkers for diagnosing this condition by further exploring the mechanisms of the kidney-gut-brain axis. This will provide a stronger theoretical basis for developing targeted therapeutic strategies.

## References

[1] Liyanage T Ninomiya T Jha V Neal B Patrice HM Okpechi I. Worldwide access to treatment for end-stage kidney disease: a systematic review. Lancet. (2015) 385:1975–82. doi: 10.1016/S0140-6736(14)61601-9, PMID: 25777665

[2] Thurlow JS Joshi M Yan G Norris KC Agodoa LY Yuan CM. Global epidemiology of end-stage kidney disease and disparities in kidney replacement therapy. Am J Nephrol. (2021) 52:98–107. doi: 10.1159/000514550, PMID: 33752206 PMC8057343

[3] Chillon J-M Massy ZA Stengel B. Neurological complications in chronic kidney disease patients. Nephrol Dial Transplant. (2016) 31:1606–14. doi: 10.1093/ndt/gfv315, PMID: 26359201

[4] Naganuma T Takemoto Y Shoji T Ishimura E Okamura M Nakatani T. Cerebral microbleeds predict intracerebral hemorrhage in hemodialysis patients. Stroke. (2015) 46:2107–12. doi: 10.1161/STROKEAHA.115.009324, PMID: 26089331

[5] Drew DA Bhadelia R Tighiouart H Novak V Scott TM Lou KV. Anatomic brain disease in hemodialysis patients: a cross-sectional study. Am J Kidney Dis. (2013) 61:271–8. doi: 10.1053/j.ajkd.2012.08.035, PMID: 23040011 PMC3546146

[6] Hsieh T-J Chang J-M Chuang H-Y Ko C-H Hsieh M-L Liu G-C. End-stage renal disease: in vivo diffusion-tensor imaging of silent White matter damage. Radiology. (2009) 252:518–25. doi: 10.1148/radiol.2523080484, PMID: 19528357

[7] Polinder-Bos HA García DV Kuipers J Elting JWJ Aries MJH Krijnen WP. Hemodialysis induces an acute decline in cerebral blood flow in elderly patients. JASN. (2018) 29:1317–25. doi: 10.1681/ASN.2017101088, PMID: 29496888 PMC5875962

[8] Chiu Y-L Tsai H-H Lai Y-J Tseng H-Y Wu Y-W Peng Y-S. Cognitive impairment in patients with end-stage renal disease: accelerated brain aging? J Formos Med Assoc. (2019) 118:867–75. doi: 10.1016/J.Jfma.2019.01.011, PMID: 30744935

[9] Olesen JB Lip GYH Kamper A-L Hommel K Køber L Lane DA. Stroke and bleeding in atrial fibrillation with chronic kidney disease. N Engl J Med. (2012) 367:625–35. doi: 10.1056/NEJMoa1105594, PMID: 22894575

[10] Chai C Wang Z Fan L Zhang M Chu Z Zuo C. Increased number and distribution of cerebral microbleeds is a risk factor for cognitive dysfunction in hemodialysis patients: a longitudinal study. Medicine. (2016) 95:E2974. doi: 10.1097/MD.0000000000002974, PMID: 27015171 PMC4998366

[11] Chen HJ Qi R Kong X Wen J Liang X Zhang Z. The impact of hemodialysis on cognitive dysfunction in patients with end-stage renal disease: a resting-state functional MRI study. Metab Brain Dis. (2015) 30:1247–56. doi: 10.1007/s11011-015-9702-0, PMID: 26146033

[12] Wu B Li X Zhang M Zhang F Long X Gong Q. Disrupted brain functional networks in patients with end-stage renal disease undergoing hemodialysis. J Neurosci Res. (2020) 98:2566–78. doi: 10.1002/jnr.24725, PMID: 32930417

[13] Sahin M Kayatas M Urun Y Sennaroglu E Akdur S. Performing only one cardiovascular reflex test has a high positive predictive value for diagnosing autonomic neuropathy in patients with chronic renal failure on hemodialysis. Ren Fail. (2006) 28:383–7. doi: 10.1080/08860220600683722, PMID: 16825086

[14] Lux S Mirzazade S Kuzmanovic B Plewan T Eickhoff SB Shah NJ. Differential activation of memory-relevant brain regions during a dialysis cycle. Kidney Int. (2010) 78:794–802. doi: 10.1038/Ki.2010.253, PMID: 20668428

[15] Murray AM Tupper DE Knopman DS Gilbertson DT Pederson SL Li S. Cognitive impairment in hemodialysis patients is common. Neurology. (2006) 67:216–23. doi: 10.1212/01.Wnl.0000225182.15532.40, PMID: 16864811

[16] Karakizlis H Bohl K Ziemek J Dodel R Hoyer J. Assessment of cognitive impairment and related risk factors in hemodialysis patients. J Nephrol. (2022) 35:931–42. doi: 10.1007/s40620-021-01170-3, PMID: 34655416 PMC8995241

[17] Gesualdo GD Duarte JG Zazzetta MS Kusumota L Say KG Pavarini SCI. Cognitive impairment of patients with chronic renal disease on hemodialysis and its relationship with sociodemographic and clinical characteristics. Dement Neuropsychol. (2017) 11:221–6. doi: 10.1590/1980-57642016dn11-030003, PMID: 29213518 PMC5674665

[18] Fadili W Al Adlouni A Louhab N Habib Allah M Kissani N Laouad I. Prevalence and risk factors of cognitive dysfunction in chronic hemodialysis patients. Aging Ment Health. (2014) 18:207–11. doi: 10.1080/13607863.2013.823375, PMID: 23906058

[19] Wolf H Grunwald M Kruggel F Riedel-Heller SG Angerhöfer S Hojjatoleslami A. Hippocampal volume discriminates between normal cognition; questionable and mild dementia in the elderly. Neurobiol Aging. (2001) 22:177–86. doi: 10.1016/S0197-4580(00)00238-4, PMID: 11182467

[20] Yaffe K Ackerson L Tamura MK Le Blanc P Kusek JW Sehgal AR. Chronic kidney disease and cognitive function in older adults: findings from the chronic renal insufficiency cohort cognitive study: chronic kidney disease and cognitive function. J Am Geriatr Soc. (2010) 58:338–45. doi: 10.1111/j.1532-5415.2009.02670.x, PMID: 20374407 PMC2852884

[21] Angermann S Schier J Baumann M Steubl D Hauser C Lorenz G. Cognitive impairment is associated with mortality in hemodialysis patients. JAD. (2018) 66:1529–37. doi: 10.3233/JAD-180767, PMID: 30412499

[22] Liu J Zhang F Wang Y Wu D. Prevalence and association of depression with uremia in dialysis population: a retrospective cohort analysis. Medicine. (2020) 99:E20401. doi: 10.1097/MD.0000000000020401, PMID: 32541461 PMC7302619

[23] Yamamoto Y Hayashino Y Akiba T Akizawa T Asano Y Saito A. Depressive symptoms predict the subsequent risk of bodily pain in dialysis patients: Japan dialysis outcomes and practice patterns study. Pain Med. (2009) 10:883–9. doi: 10.1111/j.1526-4637.2009.00661.x, PMID: 19682272

[24] Cukor D Coplan J Brown C Friedman S Cromwell-Smith A Peterson RA. Depression and anxiety in urban hemodialysis patients. Clin J Am Soc Nephrol. (2007) 2:484–90. doi: 10.2215/CJN.00040107, PMID: 17699455

[25] Cukor D Rosenthal DS Jindal RM Brown CD Kimmel PL. Depression is an important contributor to low medication adherence in hemodialyzed patients and transplant recipients. Kidney Int. (2009) 75:1223–9. doi: 10.1038/ki.2009.51, PMID: 19242502

[26] Agganis BT Weiner DE Giang LM Scott T Tighiouart H Griffith JL. Depression and cognitive function in maintenance hemodialysis patients. Am J Kidney Dis. (2010) 56:704–12. doi: 10.1053/j.ajkd.2010.04.018, PMID: 20673602 PMC2943330

[27] Pereira AA Weiner DE Scott T Sarnak MJ. Cognitive function in dialysis patients. Am J Kidney Dis. (2005) 45:448–62. doi: 10.1053/j.ajkd.2004.10.024, PMID: 15754267

[28] Li Y Zhu B Shen J Miao L. Depression in maintenance hemodialysis patients: what do we need to know? Heliyon. (2023) 9:E19383. doi: 10.1016/J.Heliyon.2023.E19383, PMID: 37662812 PMC10472011

[29] Lopes AA Bragg J Young E Goodkin D Mapes D Combe C. Depression as a predictor of mortality and hospitalization among hemodialysis patients in the United States and Europe. Kidney Int. (2002) 62:199–207. doi: 10.1046/j.1523-1755.2002.00411.x, PMID: 12081579

[30] Hedayati SS Bosworth HB Briley LP Sloane RJ Pieper CF Kimmel PL. Death or hospitalization of patients on chronic hemodialysis is associated with a physician-based diagnosis of depression. Kidney Int. (2008) 74:930–6. doi: 10.1038/ki.2008.311, PMID: 18580856

[31] Yang T Richards EM Pepine CJ Raizada MK. The gut microbiota and the brain–gut–kidney Axis in hypertension and chronic kidney disease. Nat Rev Nephrol. (2018) 14:442–56. doi: 10.1038/s41581-018-0018-2, PMID: 29760448 PMC6385605

[32] Yu-Huan S Guang-Yan C Yue-Fei X. Risk factors for intracerebral hemorrhage in patients undergoing maintenance hemodialysis. Front Neurol. (2023) 14:1111865. doi: 10.3389/Fneur.2023.1111865, PMID: 37034079 PMC10073690

[33] Bansal N Artinian NT Bakris G Chang T Cohen J Flythe J. Hypertension in patients treated with in-center maintenance hemodialysis: current evidence and future opportunities: a scientific statement from the American Heart Association. Hypertension. (2023):80. doi: 10.1161/HYP.0000000000000230 37092336

[34] Miglinas M Cesniene U Janusaite MM Vinikovas A. Cerebrovascular disease and cognition in chronic kidney disease patients. Front Cardiovasc Med. (2020) 7:96. doi: 10.3389/Fcvm.2020.00096, PMID: 32582768 PMC7283453

[35] Van Der Sande FM Hermans MMH Leunissen KML Kooman JP. Hypertension in hemodialysis patients: noncardiac consequences of hypertension in hemodialysis patients. Semin Dial. (2004) 17:304–6. doi: 10.1111/j.0894-0959.2004.17332.x, PMID: 15250923

[36] Knopman D Boland LL Mosley T Howard G Liao D Szklo M. Cardiovascular risk factors and cognitive decline in middle-aged adults. Neurology. (2001) 56:42–8. doi: 10.1212/WNL.56.1.42, PMID: 11148234

[37] Savazzi GM Cusmano F Bergamaschi E Vinci S Allegri L Garini G. Hypertension as an etiopathological factor in the development of cerebral atrophy in hemodialyzed patients. Nephron. (1999) 81:17–24. doi: 10.1159/000045240, PMID: 9884414

[38] Qian Y Zheng K Wang H You H Han F Ni J. Cerebral microbleeds and their influence on cognitive impairment in dialysis patients. Brain Imaging Behav. (2021) 15:85–95. doi: 10.1007/s11682-019-00235-z, PMID: 31898093

[39] Coladonato JA Frankenfield DL Reddan DN Klassen PS Szczech LA Johnson CA. Trends in anemia management among US hemodialysis patients. J Am Soc Nephrol. (2002) 13:1288–95. doi: 10.1097/01.asn.0000013294.11876.80, PMID: 11961017

[40] Robinson BM Joffe MM Berns JS Pisoni RL Port FK Feldman HI. Anemia and mortality in hemodialysis patients: accounting for morbidity and treatment variables updated over time. Kidney Int. (2005) 68:2323–30. doi: 10.1111/j.1523-1755.2005.00693.x, PMID: 16221236

[41] Ookawara S Ito K Sasabuchi Y Ueda Y Hayasaka H Kofuji M. Association between cerebral oxygenation, as evaluated with near-infrared spectroscopy, and cognitive function in patients undergoing hemodialysis. Nephron. (2021) 145:171–8. doi: 10.1159/000513327, PMID: 33556936

[42] Ookawara S Ito K Sasabuchi Y Hayasaka H Kofuji M Uchida T. Associations of cerebral oxygenation with hemoglobin levels evaluated by near-infrared spectroscopy in hemodialysis patients. PLoS One. (2020) 15:E0236720. doi: 10.1371/Journal.Pone.0236720, PMID: 32776946 PMC7416957

[43] Lee S-Y Lee H-J Kim Y-K Kim S-H Kim L Lee MS. Neurocognitive function and quality of life in relation to hematocrit levels in chronic hemodialysis patients. J Psychosom Res. (2004) 57:5–10. doi: 10.1016/S0022-3999(03)00528-2, PMID: 15256289

[44] Pickett JL Theberge DC Brown WS Schweitzer SU Nissenson AR. Normalizing hematocrit in dialysis patients improves brain function. Am J Kidney Dis. (1999) 33:1122–30. doi: 10.1016/S0272-6386(99)70150-2, PMID: 10352201

[45] Grimm G Stockenhuber F Schneeweiss B Madl C Zeitlhofer J Schneider B. Improvement of brain function in hemodialysis patients treated with erythropoietin. Kidney Int. (1990) 38:480–6. doi: 10.1038/ki.1990.229, PMID: 2232491

[46] Marsh JT Brown WS Wolcott D Carr CR Harper R Schweitzer SV. Rhuepo treatment improves brain and cognitive function of anemic dialysis patients. Kidney Int. (1991) 39:155–63. doi: 10.1038/ki.1991.20, PMID: 2002629

[47] Brown WS Marsh JT Wolcott D Takushi R Carr CR Higa J. Cognitive function, mood and P3 latency: effects of the amelioration of anemia in dialysis patients. Neuropsychologia. (1991) 29:35–45. doi: 10.1016/0028-3932(91)90092-m, PMID: 2017307

[48] Postiglione A Faccenda F Gallotta G Rubba P Federico S. Changes in middle cerebral artery blood velocity in uremic patients after hemodialysis. Stroke. (1991) 22:1508–11. doi: 10.1161/01.STR.22.12.1508, PMID: 1962325

[49] Murray AM Pederson SL Tupper DE Hochhalter AK Miller WA Li Q. Acute variation in cognitive function in hemodialysis patients: a cohort study with repeated measures. Am J Kidney Dis. (2007) 50:270–8. doi: 10.1053/j.ajkd.2007.05.010, PMID: 17660028

[50] Prohovnik I Post J Uribarri J Lee H Sandu O Langhoff E. Cerebrovascular effects of hemodialysis in chronic kidney disease. J Cereb Blood Flow Metab. (2007) 27:1861–9. doi: 10.1038/sj.jcbfm.9600478, PMID: 17406658

[51] Wolfgram DF. Intradialytic cerebral Hypoperfusion as mechanism for cognitive impairment in patients on hemodialysis. JASN. (2019) 30:2052–8. doi: 10.1681/ASN.2019050461, PMID: 31511363 PMC6830804

[52] Hata R Matsumoto M Handa N Terakawa H Sugitani Y Kamada T. Effects of hemodialysis on cerebral circulation evaluated by transcranial Doppler ultrasonography. Stroke. (1994) 25:408–12. doi: 10.1161/01.STR.25.2.408, PMID: 7905681

[53] Findlay MD Dawson J Dickie DA Forbes KP Mcglynn D Quinn T. Investigating the relationship between cerebral blood flow and cognitive function in hemodialysis patients. JASN. (2019) 30:147–58. doi: 10.1681/ASN.2018050462, PMID: 30530658 PMC6317612

[54] Farhoudi M Azar SA Abdi R. Brain hemodynamics in patients with end-stage renal disease between hemodialysis sessions. Iran J Kidney. (2012) 6:110–3. PMID: 22388608

[55] Mcintyre CW Goldsmith DJ. Ischemic brain injury in hemodialysis patients: which is more dangerous, hypertension or intradialytic hypotension? Kidney Int. (2015) 87:1109–15. doi: 10.1038/ki.2015.62, PMID: 25853331

[56] Eldehni MT Odudu A Mcintyre CW. Randomized clinical trial of dialysate cooling and effects on brain White matter. J Am Soc Nephrol. (2015) 26:957–65. doi: 10.1681/ASN.2013101086, PMID: 25234925 PMC4378094

[57] Chesterton LJ Selby NM Burton JO Mcintyre CW. Cool dialysate reduces asymptomatic intradialytic hypotension and increases Baroreflex variability. Hemodial Int. (2009) 13:189–96. doi: 10.1111/j.1542-4758.2009.00355.x, PMID: 19432693

[58] Libetta C Sepe V Esposito P Galli F Dal Canton A. Oxidative stress and inflammation: implications in uremia and hemodialysis. Clin Biochem. (2011) 44:1189–98. doi: 10.1016/J.Clinbiochem.2011.06.988, PMID: 21777574

[59] Belaïch R Boujraf S Housni A Maaroufi M Batta F Magoul R. Assessment of hemodialysis impact by polysulfone membrane on brain plasticity using BOLD-Fmri. Neuroscience. (2015) 288:94–104. doi: 10.1016/J.Neuroscience.2014.11.064, PMID: 25522721

[60] Muñoz-Cortés M Cabré C Villa D Vives JP Arruche M Soler J. Oxidative stress and other risk factors for White matter lesions in chronic hemodialysis patients. Clin Nephrol. (2013) 80:187–97. doi: 10.5414/CN107943, PMID: 23743154

[61] Dursun E Ozben T Süleymanlar G Dursun B Yakupoglu G. Effect of hemodialysis on the oxidative stress and antioxidants. Clin Chem Lab Med. (2002):40. doi: 10.1515/CCLM.2002.175 12476939

[62] Hörl WH. Hemodialysis membranes: interleukins, biocompatibility, and middle molecules. J Am Soc Nephrol. (2002) 13:S62–71. doi: 10.1681/ASN.V13suppl_1s62, PMID: 11792764

[63] Coombes JS Fassett RG. Antioxidant therapy in hemodialysis patients: a systematic review. Kidney Int. (2012) 81:233–46. doi: 10.1038/ki.2011.341, PMID: 21975860

[64] Jahromi SR Hosseini S Razeghi E Pasha MA Sadrzadeh H. Malnutrition predicting factors in hemodialysis patients. Saudi J Kidney Dis Transpl. (2010) 21:846–51. PMID: 20814118

[65] Massaad CA Klann E. Reactive oxygen species in the regulation of synaptic plasticity and memory. Antioxid Redox Signal. (2011) 14:2013–54. doi: 10.1089/Ars.2010.3208, PMID: 20649473 PMC3078504

[66] Himmelfarb J Stenvinkel P Ikizler TA Hakim RM. The elephant in uremia: oxidant stress as a unifying concept of cardiovascular disease in uremia. Kidney Int. (2002) 62:1524–38. doi: 10.1046/j.1523-1755.2002.00600.x, PMID: 12371953

[67] Valerianova A Lachmanova J Kovarova L Kmentova T Bartkova M Malik J. Factors responsible for cerebral hypoxia in hemodialysis population. Physiol Res. (2019) 68:651–8. doi: 10.33549/physiolres.934064, PMID: 31177793

[68] Polinder-Bos HA Elting JWJ Aries MJ García DV Willemsen AT Van Laar PJ. Changes in cerebral oxygenation and cerebral blood flow during hemodialysis – a simultaneous near-infrared spectroscopy and positron emission tomography study. J Cereb Blood Flow Metab. (2020) 40:328–40. doi: 10.1177/0271678X18818652, PMID: 30540219 PMC7370620

[69] Kovarova L Valerianova A Kmentova T Lachmanova J Hladinova Z Malik J. Low cerebral oxygenation is associated with cognitive impairment in chronic hemodialysis patients. Nephron. (2018) 139:113–9. doi: 10.1159/000487092, PMID: 29439251

[70] Schmidt R Schmidt H Curb JD Masaki K White LR Launer LJ. Early inflammation and dementia: a 25-year follow-up of the Honolulu-Asia aging study. Ann Neurol. (2002) 52:168–74. doi: 10.1002/ana.10265, PMID: 12210786

[71] Walters RJL Fox NC Crum WR Taube D Thomas DJ. Haemodialysis and cerebral oedema. Nephron. (2001) 87:143–7. doi: 10.1159/000045903, PMID: 11244309

[72] Arieff AI Massry SG Barrientos A Kleeman CR. Brain water and electrolyte metabolism in uremia: effects of slow and rapid hemodialysis. Kidney Int. (1973) 4:177–87. doi: 10.1038/ki.1973.100, PMID: 4750910

[73] Arieff AI Guisado R Massry SG Lazarowitz VC. Central nervous system Ph in uremia and the effects of hemodialysis. J Clin Invest. (1976) 58:306–11. doi: 10.1172/JCI108473, PMID: 8469 PMC333184

[74] Kumar A Cage A Dhar R. Dialysis-induced worsening of cerebral edema in intracranial hemorrhage: a case series and clinical perspective. Neurocrit Care. (2015) 22:283–7. doi: 10.1007/s12028-014-0063-z, PMID: 25228116

[75] Dalia T Tuffaha AM. Dialysis disequilibrium syndrome leading to sudden brain death in a chronic hemodialysis patient. Hemodial Int. (2018) 22:22. doi: 10.1111/Hdi.12635, PMID: 29360280

[76] Bagshaw SM Peets AD Hameed M Boiteau PJ Laupland KB Doig CJ. Dialysis disequilibrium syndrome: brain death following hemodialysis for metabolic acidosis and acute renal failure – a case report. BMC Nephrol. (2004) 5:9. doi: 10.1186/1471-2369-5-9, PMID: 15318947 PMC515303

[77] Faßbender K Mielke O Bertsch T Nafe B Fröschen S Hennerici M. Homocysteine in cerebral macroangiography and microangiopathy. Lancet. (1999) 353:1586–7. doi: 10.1016/S0140-6736(99)00309-8, PMID: 10334261

[78] Anan F Takahashi N Shimomura T Imagawa M Yufu K Nawata T. Hyperhomocysteinemia is a significant risk factor for silent cerebral infarction in patients with chronic renal failure undergoing hemodialysis. Metabolism. (2006) 55:656–61. doi: 10.1016/j.metabol.2005.12.007, PMID: 16631443

[79] Maesato K Ohtake T Mochida Y Ishioka K Oka M Moriya H. Correlation of hippocampal atrophy with Hyperhomocysteinemia in hemodialysis patients: an exploratory pilot study. PLoS One. (2017) 12:E0175102. doi: 10.1371/Journal.Pone.0175102, PMID: 28394902 PMC5386238

[80] Lipton SA Kim W-K Choi Y-B Kumar S D’Emilia DM Rayudu PV. Neurotoxicity associated with dual actions of homocysteine at the N-methyl-D-aspartate receptor. Proc Natl Acad Sci USA. (1997) 94:5923–8. doi: 10.1073/Pnas.94.11.5923, PMID: 9159176 PMC20882

[81] Parnetti L Bottiglieri T Lowenthal D. Role of homocysteine in age-related vascular and non-vascular diseases. Aging Clin Exp Res. (1997) 9:241–57. doi: 10.1007/BF03341827, PMID: 9359935

[82] Bachmann J Tepel M Raidt H Riezler R Graefe U Langer K. Hyperhomocysteinemia and the risk for vascular disease in hemodialysis patients. J Am Soc Nephrol. (1995) 6:121–5. doi: 10.1681/ASN.V61121, PMID: 7579064

[83] Suliman ME Qureshi AR Bárány P Stenvinkel P Filho JCD Anderstam B. Hyperhomocysteinemia, nutritional status, and cardiovascular disease in hemodialysis patients. Kidney Int. (2000) 57:1727–35. doi: 10.1046/j.1523-1755.2000.00018.x, PMID: 10760109

[84] Jorde R Waterloo K Saleh F Haug E Svartberg J. Neuropsychological function in relation to serum parathyroid hormone and serum 25–hydroxyvitamin D levels: the Tromsø study. J Neurol. (2006) 253:464–70. doi: 10.1007/s00415-005-0027-5, PMID: 16283099

[85] Diskin J Diskin CJ. Mental effects of excess parathyroid hormone in hemodialysis patients: a possible role for parathyroid 2 hormone receptor? Ther Apher Dial. (2020) 24:285–9. doi: 10.1111/1744-9987.13429, PMID: 31423747

[86] Cogan MG Covey CM Arieff AI Wisniewski A Clark OH. Central nervous system manifestations of hyperparathyroidism. Am J Med. (1978) 65:963–70. doi: 10.1016/0002-9343(78)90748-9, PMID: 742632

[87] Herrington W Haynes R Staplin N Emberson J Baigent C Landray M. Evidence for the prevention and treatment of stroke in dialysis patients. Semin Dial. (2015) 28:35–47. doi: 10.1111/sdi.12281, PMID: 25040468 PMC4320775

[88] Tagawa M Hamano T Nishi H Tsuchida K Hanafusa N Fukatsu A. Mineral metabolism markers are associated with myocardial infarction and hemorrhagic stroke but not ischemic stroke in hemodialysis patients: a longitudinal study. PLoS One. (2014) 9:E114678. doi: 10.1371/Journal.Pone.0114678, PMID: 25494334 PMC4262415

[89] Tanaka M Yamazaki S Hayashino Y Fukuhara S Akiba T Saito A. Hypercalcaemia is associated with poor mental health in Haemodialysis patients: results from Japan DOPPS. Nephrol Dial Transplant. (2007) 22:1658–64. doi: 10.1093/ndt/gfm008, PMID: 17298993

[90] Gallieni M Brancaccio D Padovese P Rolla D Bedani P Colantonio G. On behalf of the Italian group for the study of intravenous calcitriol. Low-dose intravenous calcitriol treatment of secondary hyperparathyroidism in hemodialysis patients. Kidney Int. (1992) 42:1191–8. doi: 10.1038/ki.1992.404, PMID: 1453603

[91] Malberti F Corradi B Cosci P Calliad F Marcelli D Imbasciati E. Long-term effects of intravenous calcitriol therapy on the control of secondary hyperparathyroidism. Am J Kidney Dis. (1996) 28:704–12. doi: 10.1016/S0272-6386(96)90252-8, PMID: 9158208

[92] Cooper JD Lazarowitz VC Arieff AI. Neurodiagnostic abnormalities in patients with acute renal failure. J Clin Invest. (1978) 61:1448–55. doi: 10.1172/JCI109064, PMID: 659607 PMC372670

[93] Zhang J Hu J Zhou R Xu Y. Cognitive function and vitamin D status in the Chinese hemodialysis patients. Comput Math Methods Med. (2022) 2022:1–6. doi: 10.1155/2022/2175020, PMID: 36118837 PMC9481383

[94] Yavuz D Demirağ MD Yavuz R Karagöz Özen DS Ramazanoğlu ZB. 25-Hydroxy vitamin D level is associated with sleep disturbances in patients with chronic kidney disease on hemodialysis: a cross-sectional study. Turk J Med Sci. (2020) 44:298–303. doi: 10.3906/sag-1908-87, PMID: 31887852 PMC7164765

[95] Shaffi K Tighiouart H Scott T Lou K Drew D Weiner D. Low 25-Hydroxyvitamin D levels and cognitive impairment in hemodialysis patients. Clin J Am Soc Nephrol. (2013) 8:979–86. doi: 10.2215/CJN.10651012, PMID: 23449769 PMC3675858

[96] Ritz E. Intestinal-renal syndrome: mirage or reality? Blood Purif. (2011) 31:70–6. doi: 10.1159/000321848, PMID: 21228570

[97] Evenepoel P Poesen R Meijers B. The gut–kidney Axis. Pediatr Nephrol. (2017) 32:2005–14. doi: 10.1007/s00467-016-3527-x, PMID: 27848096

[98] Jandhyala SM. Role of the normal gut microbiota. WJG. (2015) 21:8787–803. doi: 10.3748/wjg.v21.i29.8787, PMID: 26269668 PMC4528021

[99] Guarner F Malagelada JR. Gut Flora in health and disease. Lancet. (2003) 361:512–9. doi: 10.1016/S0140-6736(03)12489-0, PMID: 12583961

[100] Sonnenburg JL Angenent LT Gordon JI. Getting a grip on things: how do communities of bacterial symbionts become established in our intestine? Nat Immunol. (2004) 5:569–73. doi: 10.1038/ni1079, PMID: 15164016

[101] Felizardo RJF Castoldi A Andrade-Oliveira V Câmara NOS. The microbiota and chronic kidney diseases: a double-edged sword. Clin Trans Imm. (2016) 5:E86. doi: 10.1038/Cti.2016.36, PMID: 27757226 PMC5067952

[102] Hugon P Dufour J-C Colson P Fournier P-E Sallah K Raoult D. A comprehensive repertoire of prokaryotic species identified in human beings. Lancet Infect Dis. (2015) 15:1211–9. doi: 10.1016/S1473-3099(15)00293-5, PMID: 26311042

[103] Eckburg PB Bik EM Bernstein CN Purdom E Dethlefsen L Sargent M. Diversity of the human intestinal microbial Flora. Science. (2005) 308:1635–8. doi: 10.1126/science.1110591, PMID: 15831718 PMC1395357

[104] Shi K Wang F Jiang H Liu H Wei M Wang Z. Gut bacterial translocation may aggravate microinflammation in hemodialysis patients. Dig Dis Sci. (2014) 59:2109–17. doi: 10.1007/s10620-014-3202-7, PMID: 24828917

[105] Bossola M Sanguinetti M Scribano D Zuppi C Giungi S Luciani G. Circulating bacterial-derived DNA fragments and markers of inflammation in chronic hemodialysis patients. Clin J Am Soc Nephrol. (2009) 4:379–85. doi: 10.2215/CJN.03490708, PMID: 19118119 PMC2637587

[106] He H Xie Y. Effect of different hemodialysis methods on microbiota in uremic patients. Biomed Res Int. (2020) 2020:1–8. doi: 10.1155/2020/6739762, PMID: 32685517 PMC7321504

[107] Wu H-Y Lin Y-T Tsai W-C Chiu Y-L Ko M-J Yang J-Y. Microbiota analysis in the hemodialysis population-focusing on enterobacteriaceae. J Microbiol Immunol Infect. (2023) 56:311–23. doi: 10.1016/j.jmii.2022.12.001, PMID: 36535841

[108] Zhang X Wang Y Yin Y Sun B Chen G Chen F. Changes of gut microbiota in maintenance hemodialysis patients and their impact on patient’s microinflammation status. Cell Mol Biol. (2023) 69:96–101. doi: 10.14715/cmb/2023.69.8.15, PMID: 37715414

[109] Chao YT Lin Y-K Chen L-K Huang P Hsu Y-C. Role of the gut microbiota and their metabolites in hemodialysis patients. Int J Med Sci. (2023) 20:725–36. doi: 10.7150/ijms.82667, PMID: 37213669 PMC10198149

[110] Pan X Raaijmakers JM Carrión VJ. Importance of Bacteroidetes in host-microbe interactions and ecosystem functioning. Trends Microbiol. (2023) 31:959–71. doi: 10.1016/j.tim.2023.03.018, PMID: 37173204

[111] Vaziri ND Yuan J Rahimi A Ni Z Said H Subramanian VS. Disintegration of colonic epithelial tight junction in uremia: a likely cause of CKD-associated inflammation. Nephrol Dial Transplant. (2012) 27:2686–93. doi: 10.1093/ndt/gfr624, PMID: 22131233 PMC3616758

[112] Vaziri ND. CKD impairs barrier function and alters microbial Flora of the intestine: a major link to inflammation and uremic toxicity. Curr Opin Nephrol Hypertens. (2012) 21:587–92. doi: 10.1097/MNH.0b013e328358c8d5, PMID: 23010760 PMC3756830

[113] Flobert C Cellier C Berger A Ngo A Cuillerier E Landi B. Right colonic involvement is associated with severe forms of ischemic colitis and occurs frequently in patients with chronic renal failure requiring hemodialysis. Am J Gastroenterol. (2000) 95:195–8. doi: 10.1111/j.1572-0241.2000.01644.x, PMID: 10638582

[114] Navarro JF Mora C León C Martín-Del Río R Macía ML Gallego E. Amino acid losses during hemodialysis with polyacrylonitrile membranes: effect of intradialytic amino acid supplementation on plasma amino acid concentrations and nutritional variables in nondiabetic patients. Am J Clin Nutr. (2000) 71:765–73. doi: 10.1093/ajcn/71.3.765, PMID: 10702171

[115] Lin T-Y Hung S-C. Association of subjective global assessment of nutritional status with gut microbiota in hemodialysis patients: a case-control study. Nephrol Dial Transplant. (2021) 36:1104–11. doi: 10.1093/ndt/gfaa019, PMID: 32240309

[116] Cao C Zhu H Yao Y Zeng R. Gut Dysbiosis and kidney diseases. Front Med. (2022) 9:9. doi: 10.3389/Fmed.2022.829349, PMID: 35308555 PMC8927813

[117] Chen Y Xu J Chen Y. Regulation of neurotransmitters by the gut microbiota and effects on cognition in neurological disorders. Nutrients. (2021) 13:2099. doi: 10.3390/nu13062099, PMID: 34205336 PMC8234057

[118] Cryan JF O’Riordan KJ Sandhu K Peterson V Dinan TG. The gut microbiome in neurological disorders. Lancet Neurol. (2020) 19:179–94. doi: 10.1016/S1474-4422(19)30356-4, PMID: 31753762

[119] Zhu B Shen J Jiang R Jin L Zhan G Liu J. Abnormalities in gut microbiota and serum metabolites in hemodialysis patients with mild cognitive decline: a single-center observational study. Psychopharmacology. (2020) 237:2739–52. doi: 10.1007/s00213-020-05569-x, PMID: 32601991

[120] Stenvinkel P. Inflammation in end-stage renal failure: could it be treated? Nephrol Dial Transplant. (2002) 17:33–8. doi: 10.1093/ndt/17.suppl_8.33, PMID: 12147775

[121] Kotanko P Carter M Levin NW. Intestinal bacterial microflora—a potential source of chronic inflammation in patients with chronic kidney disease. Nephrol Dial Transplant. (2006) 21:2057–60. doi: 10.1093/ndt/gfl281, PMID: 16762961

[122] Wang YF Zheng LJ Liu Y Ye YB Luo S Lu GM. The gut microbiota-inflammation-brain axis in end-stage renal disease: perspectives from default mode network. Theranostics. (2019) 9:8171–81. doi: 10.7150/thno.35387, PMID: 31754388 PMC6857049

[123] Zimmermann J Herrlinger S Pruy A Metzger T Wanner C. Inflammation enhances cardiovascular risk and mortality in hemodialysis patients. Kidney Int. (1999) 55:648–58. doi: 10.1046/j.1523-1755.1999.00273.x, PMID: 9987089

[124] Arici M Walls J. End-stage renal disease, atherosclerosis, and cardiovascular mortality: is C-reactive protein the missing link? Kidney Int. (2001) 59:407–14. doi: 10.1046/j.1523-1755.2001.059002407.x, PMID: 11168922

[125] Papagianni A. Carotid atherosclerosis is associated with inflammation and endothelial cell adhesion molecules in chronic haemodialysis patients. Nephrol Dial Transplant. (2003) 18:113–9. doi: 10.1093/ndt/18.1.113, PMID: 12480968

[126] Fanadka F Rozenberg I Nacasch N Einbinder Y Benchetrit S Wand O. Intra-cranial arterial calcifications in hemodialysis patients. Medicina (Kaunas). (2023) 59:1706. doi: 10.3390/medicina59101706, PMID: 37893424 PMC10608215

[127] Anan F Shimomura T Kaku T Kaneda K Imagawa M Tsukagawa H. High-sensitivity C-reactive protein level is a significant risk factor for silent cerebral infarction in patients on hemodialysis. Metabolism. (2008) 57:66–70. doi: 10.1016/j.metabol.2007.08.007, PMID: 18078860

[128] Ng A Tam WW Zhang MW Ho CS Husain SF Mcintyre RS. IL-1β, IL-6, TNF-Α and CRP in elderly patients with depression or Alzheimer’s disease: systematic review and meta-analysis. Sci Rep. (2018) 8:12050. doi: 10.1038/S41598-018-30487-6, PMID: 30104698 PMC6089986

[129] Cattaneo A Cattane N Galluzzi S Provasi S Lopizzo N Festari C. Association of brain amyloidosis with pro-inflammatory gut bacterial taxa and peripheral inflammation markers in cognitively impaired elderly. Neurobiol Aging. (2017) 49:60–8. doi: 10.1016/j.neurobiolaging.2016.08.019, PMID: 27776263

[130] Liang X Fu Y Cao W Wang Z Zhang K Jiang Z. Gut microbiome, cognitive function and brain structure: a multi-omics integration analysis. Transl Neurodegener. (2022) 11:49. doi: 10.1186/S40035-022-00323-Z, PMID: 36376937 PMC9661756

[131] Maesaka JK Palaia T Fishbane S Ragolia L. Contribution of prostaglandin D2 synthase to progression of renal failure and dialysis dementia. Semin Nephrol. (2002) 22:407–14. doi: 10.1053/snep.2002.34726, PMID: 12224048

[132] Bossola M Ciciarelli C Conte GL Vulpio C Luciani G Tazza L. Correlates of symptoms of depression and anxiety in chronic hemodialysis patients. Gen Hosp Psychiatry. (2010) 32:125–31. doi: 10.1016/j.genhosppsych.2009.10.009, PMID: 20302985

[133] Stinghen AEM Pecoits-Filho R. Vascular damage in kidney disease: beyond hypertension. Int J Hypertens. (2011) 2011:1–5. doi: 10.4061/2011/232683, PMID: 21876786 PMC3160729

[134] Lin Y-T Wu P-H Liang S-S Mubanga M Yang Y-H Hsu Y-L. Protein-bound uremic toxins are associated with cognitive function among patients undergoing maintenance hemodialysis. Sci Rep. (2019) 9:20388. doi: 10.1038/S41598-019-57004-7, PMID: 31892730 PMC6938492

[135] Lin Y-T Wu P-H Lee H-H Mubanga M Chen C-S Kuo M-C. Indole-3 acetic acid increased risk of impaired cognitive function in patients receiving hemodialysis. Neurotoxicology. (2019) 73:85–91. doi: 10.1016/j.neuro.2019.02.019, PMID: 30826344

[136] Sun C-Y Li J-R Wang Y-Y Lin S-Y Ou Y-C Lin C-J. P-cresol sulfate caused behavior disorders and neurodegeneration in mice with unilateral nephrectomy involving oxidative stress and neuroinflammation. IJMS. (2020) 21:6687. doi: 10.3390/ijms21186687, PMID: 32932690 PMC7555291

[137] Liabeuf S Pepin M Franssen CFM Viggiano D Carriazo S Gansevoort RT. Chronic kidney disease and neurological disorders: are Uraemic toxins the missing piece of the puzzle? Nephrol Dial Transplant. (2021) 37:ii33–44. doi: 10.1093/ndt/gfab223, PMID: 34718753 PMC8713157

[138] De Deyn PP Vanholder R Eloot S Glorieux G. Progress in uremic toxin research: Guanidino compounds as uremic (neuro)toxins. Semin Dial. (2009) 22:340–5. doi: 10.1111/j.1525-139X.2009.00577.x, PMID: 19708978

[139] Faucher Q Van Der Made TK De Lange E Masereeuw R. Blood-brain barrier perturbations by uremic toxins: key contributors in chronic kidney disease-induced neurological disorders? Eur J Pharm Sci. (2023) 187:106462. doi: 10.1016/j.ejps.2023.106462, PMID: 37169097

[140] Assem M Lando M Grissi M Kamel S Massy Z Chillon J-M. The impact of uremic toxins on cerebrovascular and cognitive disorders. Toxins. (2018) 10:303. doi: 10.3390/toxins10070303, PMID: 30037144 PMC6071092

[141] Oshima N Onimaru H Matsubara H Uchida T Watanabe A Takechi H. Uric acid, Indoxyl sulfate, and Methylguanidine activate Bulbospinal neurons in the RVLM via their specific transporters and by producing oxidative stress. Neuroscience. (2015) 304:133–45. doi: 10.1016/j.neuroscience.2015.07.055, PMID: 26208844

[142] Watanabe K Sato E Mishima E Watanabe M Abe T Takahashi N. Effect of uremic toxins on hippocampal cell damage: analysis in vitro and in rat model of chronic kidney disease. Heliyon. (2021) 7:E06221. doi: 10.1016/J.Heliyon.2021.E06221, PMID: 33659745 PMC7892929

[143] Bobot M Thomas L Moyon A Fernandez S Mckay N Balasse L. Uremic toxic blood-brain barrier disruption mediated by Ahr activation leads to cognitive impairment during experimental renal dysfunction. JASN. (2020) 31:1509–21. doi: 10.1681/ASN.2019070728, PMID: 32527975 PMC7350986

[144] Hamed SA. Neurologic conditions and disorders of uremic syndrome of chronic kidney disease: presentations, causes, and treatment strategies. Expert Rev Clin Pharmacol. (2019) 12:61–90. doi: 10.1080/17512433.2019.1555468, PMID: 30501441

[145] Lin Y-T Wu P-H Tsai Y-C Hsu Y-L Wang HY Kuo M-C. Indoxyl sulfate induces apoptosis through oxidative stress and mitogen-activated protein kinase signaling pathway inhibition in human astrocytes. JCM. (2019) 8:191. doi: 10.3390/jcm8020191, PMID: 30764571 PMC6406290

[146] Deguchi T Isozaki K Yousuke K Terasaki T Otagiri M. Involvement of organic anion transporters in the efflux of uremic toxins across the blood–brain barrier. J Neurochem. (2006) 96:1051–9. doi: 10.1111/j.1471-4159.2005.03550.x, PMID: 16445853

[147] Fang C Lau WL Sun J Chang R Vallejo A Lee D. Chronic kidney disease promotes cerebral microhemorrhage formation. J Neuroinflammation. (2023) 20:51. doi: 10.1186/S12974-023-02703-2, PMID: 36841828 PMC9960195

[148] Baumgaertel MW Kraemer M Berlit P. Neurologic complications of acute and chronic renal disease In: Handbook of clinical neurology. Eds. Biller J and Ferro JM Elsevier (2014) 119:383–93. doi: 10.1016/B978-0-7020-4086-3.00024-2 24365307

[149] Adesso S Magnus T Cuzzocrea S Campolo M Rissiek B Paciello O. Indoxyl sulfate affects glial function increasing oxidative stress and neuroinflammation in chronic kidney disease: interaction between astrocytes and microglia. Front Pharmacol. (2017) 8:370. doi: 10.3389/Fphar.2017.00370, PMID: 28659803 PMC5466960

[150] Wu P-H Tseng Y-F Liu W Chuang Y-S Tai C-J Tung C-W. Exploring the relationship between gut microbiome composition and blood Indole-3-acetic acid in hemodialysis patients. Biomedicine. (2024) 12:148. doi: 10.3390/biomedicines12010148, PMID: 38255253 PMC10813781

[151] Karu N Mckercher C Nichols DS Davies N Shellie RA Hilder EF. Tryptophan metabolism, its relation to inflammation and stress markers and association with psychological and cognitive functioning: Tasmanian chronic kidney disease pilot study. BMC Nephrol. (2016) 17:171. doi: 10.1186/S12882-016-0387-3, PMID: 27832762 PMC5103367

[152] Mcintyre CW Harrison LEA Eldehni MT Jefferies HJ Szeto C-C John SG. Circulating Endotoxemia: a novel factor in systemic inflammation and cardiovascular disease in chronic kidney disease. Clin J Am Soc Nephrol. (2011) 6:133–41. doi: 10.2215/CJN.04610510, PMID: 20876680 PMC3022234

[153] Sherwin E Dinan TG Cryan JF. Recent developments in understanding the role of the gut microbiota in brain health and disease. Ann N Y Acad Sci. (2018) 1420:5–25. doi: 10.1111/Nyas.13416, PMID: 28768369

[154] Strandwitz P Kim KH Terekhova D Liu JK Sharma A Levering J. GABA-modulating Bacteria of the human gut microbiota. Nat Microbiol. (2018) 4:396–403. doi: 10.1038/S41564-018-0307-3, PMID: 30531975 PMC6384127

[155] Strandwitz P. Neurotransmitter modulation by the gut microbiota. Brain Res. (2018) 1693:128–33. doi: 10.1016/j.brainres.2018.03.015, PMID: 29903615 PMC6005194

[156] Qu S Yu Z Zhou Y Wang S Jia M Chen T. Gut microbiota modulates neurotransmitter and gut-brain signaling. Microbiol Res. (2024) 287:127858. doi: 10.1016/j.micres.2024.127858, PMID: 39106786

[157] Kaelberer MM Buchanan KL Klein ME Barth BB Montoya MM Shen X. A gut-brain neural circuit for nutrient sensory transduction. Sens Transduct. (2018) 361:Eaat5236. doi: 10.1126/Science.Aat5236, PMID: 30237325 PMC6417812

[158] Cox LM Weiner HL. Microbiota signaling pathways that influence neurologic disease. Neurotherapeutics. (2018) 15:135–45. doi: 10.1007/s13311-017-0598-8, PMID: 29340928 PMC5794708

[159] Sweeney VP Perry TL Price JD Reeve CE Godolphin WJ Kish SJ. Brain gamma-aminobutyric acid deficiency in dialysis encephalopathy. Neurology. (1985) 35:180–4. doi: 10.1212/WNL.35.2.180, PMID: 3969205

[160] Perry TL Yong VW Kish SJ Ito M Foulks JG Godolphin WJ. Neurochemical abnormalities in brains of renal failure patients treated by repeated hemodialysis. J Neurochem. (1985) 45:1043–8. doi: 10.1111/j.1471-4159.1985.tb05521.x, PMID: 2411864

[161] Cooper TE Khalid R Chan S Craig JC Hawley CM Howell M. Synbiotics, prebiotics and probiotics for people with chronic kidney disease. Cochrane Database Syst Rev. (2023) 10:CD013631. doi: 10.1002/14651858.CD013631.Pub2, PMID: 37870148 PMC10591284

[162] Du J Zhao X Ding X Han Q Duan Y Ren Q. The role of the gut microbiota in complications among hemodialysis patients. Microorganisms. (2024) 12:1878. doi: 10.3390/microorganisms12091878, PMID: 39338552 PMC11434415

[163] Haghighat N Mohammadshahi M Shayanpour S Haghighizadeh MH. Effects of synbiotics and probiotics supplementation on serum levels of endotoxin, heat shock protein 70 antibodies and inflammatory markers in hemodialysis patients: a randomized double-blinded controlled trial. Probiotics Antimicrob Proteins. (2020) 12:144–51. doi: 10.1007/s12602-018-9509-5, PMID: 30617950

[164] Nguyen TTU Kim HW Kim W. Effects of probiotics, prebiotics, and Synbiotics on uremic toxins, inflammation, and oxidative stress in hemodialysis patients: a systematic review and Meta-analysis of randomized controlled trials. JCM. (2021) 10:4456. doi: 10.3390/jcm10194456, PMID: 34640474 PMC8509328

